# Femtosecond X-ray diffraction from an aerosolized beam of protein nanocrystals

**DOI:** 10.1107/S1600576717018131

**Published:** 2018-02-01

**Authors:** Salah Awel, Richard A. Kirian, Max O. Wiedorn, Kenneth R. Beyerlein, Nils Roth, Daniel A. Horke, Dominik Oberthür, Juraj Knoska, Valerio Mariani, Andrew Morgan, Luigi Adriano, Alexandra Tolstikova, P. Lourdu Xavier, Oleksandr Yefanov, Andrew Aquila, Anton Barty, Shatabdi Roy-Chowdhury, Mark S. Hunter, Daniel James, Joseph S. Robinson, Uwe Weierstall, Andrei V. Rode, Saša Bajt, Jochen Küpper, Henry N. Chapman

**Affiliations:** a Center for Free-Electron Laser Science, Deutsches Elektronen-Synchrotron DESY, Notkestrasse 85, 22607 Hamburg, Germany; b The Hamburg Center for Ultrafast Imaging, Universität Hamburg, Luruper Chaussee 149, 22761 Hamburg, Germany; c Arizona State University, Tempe, Arizona, USA; d Department of Physics, Universität Hamburg, Luruper Chaussee 149, 22761 Hamburg, Germany; e Photon Science, Deutsches Elektronen-Synchrotron DESY, Notkestrasse 85, 22607 Hamburg, Germany; f Max-Planck Institute for the Structure and Dynamics of Matter, Luruper Chaussee 149, 22761 Hamburg, Germany; g Linac Coherent Light Source (LCLS), SLAC National Accelerator Laboratory, 2575 Sand Hill Road, Menlo Park, CA 94025, USA; h Laser Physics Centre, Research School of Physics and Engineering, Australian National University, ACT 2601, Canberra, Australia

**Keywords:** X-ray diffraction, aerosols, nanocrystals

## Abstract

High-resolution Bragg diffraction from aerosolized single granulovirus nanocrystals using an X-ray free-electron laser is demonstrated. The outer dimensions of the in-vacuum aerosol injector components are identical to conventional liquid-microjet nozzles used in serial diffraction experiments, which allows the injector to be utilized with standard mountings. As compared with liquid-jet injection, the X-ray scattering background is reduced by several orders of magnitude by the use of helium carrier gas rather than liquid. Such reduction is required for diffraction measurements of small macromolecular nanocrystals and single particles. High particle speeds are achieved, making the approach suitable for use at upcoming high-repetition-rate facilities.

## Abstract 2

A new particle-injection approach is demonstrated that achieves very low background in the measurement of diffraction from macromolecular nanocrystals by using an aerosol-focusing injector with an X-ray free-electron laser.

## 1. Introduction   

Serial femtosecond crystallography (SFX) allows the structural analysis of macromolecular crystals that may be too small or weakly scattering to study with synchrotron radiation sources. In order to record any measurable diffraction signal, such samples would require exposures far in excess of limits imposed by X-ray-induced radiation damage when using conventional sources. With typical pulse energies of about 1 mJ, or 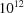 photons, and durations of about 10 fs, pulses from X-ray free-electron lasers (XFELs) overcome this limit by producing diffraction data before the onset of most damage processes (Neutze *et al.*, 2000 ▸; Chapman *et al.*, 2011 ▸; Boutet *et al.*, 2012 ▸). Furthermore, XFELs enable novel time-resolved studies with femtosecond temporal resolution and ångström spatial resolution, all at physiological temperatures. A variety of prominent results from SFX measurements are summarized in recent reviews and special issues (Spence *et al.*, 2012 ▸; Schlichting & Miao, 2012 ▸; Barty *et al.*, 2013 ▸; Patterson, 2014 ▸; Schlichting, 2015 ▸; Johansson *et al.*, 2017 ▸).

The large increase in X-ray fluence afforded by the ability to outrun damage not only increases the diffraction signal from the sample but also increases the diffuse scattering from the medium transporting the crystal to the beam. Many SFX measurements were, therefore, performed on microcrystals that were large enough and ordered well enough to produce intense Bragg reflections that could be measured in the presence of the diffuse background. Such examples helped the rapid adoption of the technique. The use of such crystals, usually with volumes greater than 1 µm^3^, enabled a broad range of sample delivery methods to be used depending on the nature of the experiment, such as liquid microjets (DePonte *et al.*, 2008 ▸; Weierstall, 2014 ▸), viscous extrusion injectors (Weierstall *et al.*, 2014 ▸) and solid substrates (Frank *et al.*, 2014 ▸; Roedig *et al.*, 2017 ▸). This flexibility is in stark contrast to efforts to record high-resolution coherent diffraction patterns from noncrystalline samples (Seibert *et al.*, 2011 ▸; Küpper *et al.*, 2014 ▸; Aquila *et al.*, 2015 ▸; Yang *et al.*, 2016 ▸). Without the amplification of the diffraction signal due to periodicity, objects such as molecules, viruses and other particles produce only weak scattering signals. Noncrystalline samples must, therefore, be delivered to the X-ray focus in a vacuum environment and in isolation from other potential scattering sources. This can be achieved, for example, through aerodynamic focusing of aerosolized particles (Bogan *et al.*, 2008 ▸; Bogan, Boutet *et al.*, 2010 ▸; Bogan, Starodub *et al.*, 2010 ▸; Roth *et al.*, 2017 ▸). In certain cases, background scattering from a dense surrounding medium is highly undesirable even for experiments on crystalline samples. For example, imaging techniques have been developed to utilize the faint continuous diffraction signal in regions between and at scattering angles beyond the intense Bragg reflections due to lattice disorder (Ayyer *et al.*, 2016 ▸; Chapman *et al.*, 2017 ▸) or lattice truncation (Spence *et al.*, 2011 ▸; Kirian, Bean *et al.*, 2015 ▸). The advantage and motivation for such approaches is that the continuous diffraction that can be accessed provides a direct route to solving the crystallographic phase problem without the need for prior knowledge or additional measurements.

Here, we demonstrate high-resolution X-ray diffraction from isolated protein nanocrystals delivered into the XFEL focus *via* a convergent-nozzle aerosol injector (CNAI) (Kirian, Awel *et al.*, 2015 ▸). We show that the aerosol delivery produces extremely low background scattering signals compared with a conventional liquid jet. This aerosol injector has essentially the same size and form as the nozzles that are commonly used to produce liquid jets for SFX experiments (DePonte *et al.*, 2008 ▸; Beyerlein *et al.*, 2015 ▸; Oberthuer *et al.*, 2017 ▸) and hence can be installed using standard liquid-jet mountings available at X-ray facilities. As shown in our previous work, CNAIs can produce aerosolized beams of sub-micrometre particles with a full-width at half maximum diameter <5 µm and particle speeds of the order of a few hundred metres per second, depending on particle size and operating conditions. This high velocity may be well suited to the megahertz repetition rates of upcoming XFEL sources.

## 2. Experimental methods   

This proof-of-principle experiment was performed on natural *Cydia pomonella* granulovirus (GV) particles of approximately 200 × 200 × 370 nm in size that consist of a central virus body surrounded by a crystalline granulin protein shell. They infect invertebrates such as the codling moth (*Cydia pomonella*) (Jehle *et al.*, 2006 ▸). The GV particles used in this study were purified from a biopesticide solution (Certis Madex HP) using a method described elsewhere (Oberthuer *et al.*, 2017 ▸) and suspended in water at a concentration of approximately 3 × 10^11^ particles ml^−1^ prior to injection. The particle concentration was measured using a NanoSight (model LM14C) particle analysis system. The volume of the particle is about 0.015 µm^3^, with about 2/3 of that found as the volume of the crystalline shell (Gati *et al.*, 2017 ▸), which corresponds to a diameter of approximately 300 nm for a sphere of equivalent volume. Despite the small size of these particles, previous SFX experiments recorded diffraction to 2.1 Å resolution from such nanocrystals delivered to the X-ray beam in a gas-focused liquid jet (Gati *et al.*, 2017 ▸).

Diffraction measurements were performed in the nanofocus chamber at the coherent X-ray imaging (CXI) (Liang *et al.*, 2015 ▸) instrument at the Linac Coherent Light Source (LCLS). The experiment was carried out immediately after a successful liquid-jet experiment (Oberthuer *et al.*, 2017 ▸) without disruption to the X-ray beam. During that earlier experiment the beam focus was optimized by adjusting the Kirkpatrick–Baez focusing mirrors *via* analysis of spot imprints on a gold foil. After optimization, the position of the beam was determined by placing an yttrium aluminum garnet (YAG) screen in the focal plane and observing optical fluorescence with a fixed in-line microscope with a resolution of a few micrometres. In our experiment, the aerosol beam was initially aligned relative to this reference and then scanned in position as described below.

**Figure 1:**
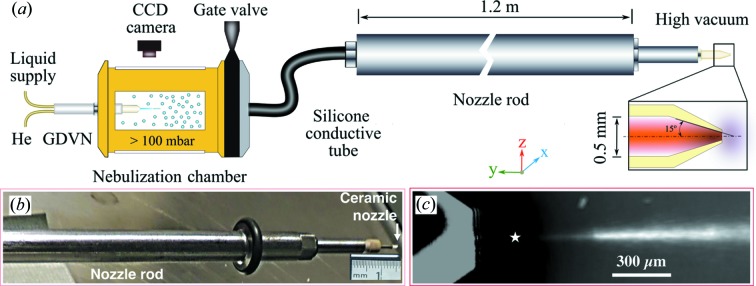
CNAI assembly and its operation during the CXI experiment. (*a*) Sketch of the basic aerosol generation and transportation setup. (*b*) The aerosol nozzle mounted on the nozzle rod. (*c*) Time-integrated image of a laser-illuminated stream of GV particles exiting the CNAI, recorded using the in-line microscope at the CXI instrument. This image was formed by averaging over 3.7 min, with a running median background subtracted from each frame. The CNAI tip is seen in the left portion of the image, and the approximate X-ray focal point is indicated by the star.

The granulovirus suspension was aerosolized using a gas dynamic virtual nozzle (GDVN) (Beyerlein *et al.*, 2015 ▸) mounted in a cylindrical nebulization chamber as depicted in Fig. 1 ▸(*a*). A GDVN uses gas-flow focusing to create a liquid jet with a diameter significantly smaller than the orifice of the nozzle, and which consequently breaks up to form a mist of droplets. The liquid was pressurized to flow from the nozzle at rates between 2.7 and 3.5 µl min^−1^, producing droplets of about 2 µm diameter at a rate between 11 × 10^6^ and 14 × 10^6^ s^−1^, each containing on average 1.3 nanocrystals. This is equivalent to particle flow rates of 8.6 × 10^8^–1.1 × 10^9^ particles min^−1^.

The focusing gas was helium, which was set to a mass flow rate in the range of 10–60 mg min^−1^. The nebulization chamber had an inner diameter of approximately 40 mm and was 150 mm in length, giving a residence time in the chamber of several minutes, and a helium pressure that stabilized at a value between 100 mbar and 1 bar (1 bar = 10^5^ Pa). Under these conditions most of the solvent evaporated to produce nanocrystals suspended in a humid helium atmosphere (Kirian, Awel *et al.*, 2015 ▸). Drops that contain more than one particle during the initial stage most likely form clusters of crystals (Cho *et al.*, 2007 ▸; Daurer *et al.*, 2017 ▸). The aerosol flowed through conductive silicone rubber tubing (Simolex, 6.3 mm inner diameter, 30 cm length), which was coupled to a standard ‘nozzle rod’ of the CXI beamline. This is a 1.2 m long stainless steel tube with a 6.3 mm inner diameter that is normally used to transfer liquid-jet injectors in and out of the main experimental chamber without breaking vacuum (Weierstall *et al.*, 2012 ▸). The conductive tubing along the entire particle path acted as a Faraday cage to shield external electric fields from interacting with particles that might become charged through triboelectric effects in the GDVN. The aerosol finally exited the CNAI, which was mounted at the end of the CXI nozzle rod much like a typical liquid-jet nozzle. It consisted of a ceramic injection-molded tube of 1 mm outer diameter and 500 µm inner diameter, a short converging section with a convergence angle of 15°, and a 100 µm exit aperture (further details can be found in our previous work; Kirian, Awel *et al.*, 2015 ▸).

**Figure 2:**
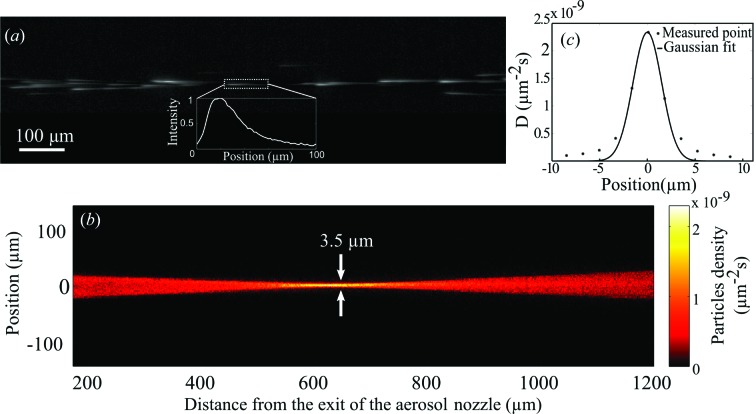
Laboratory characterization of a beam of GV particles focused with the 15° convergent aerosol nozzle using a strong-magnification imaging microscope. (*a*) A single exposure, showing streaked images of GV particles caused by the 100 ns laser illumination. The particles are moving from left to right and their streaked images have non-uniform intensity due to the relatively slow decay of the illumination laser pulses. (*b*) The two-dimensional rate-corrected particle density determined from the centroids of individual particle images such as the one shown in (*a*). (*c*) Gaussian fit to the particle density at the focal plane in (*b*).

During the diffraction experiment we monitored the crystal injection through direct optical imaging of scattered laser light from injected particles (Awel *et al.*, 2016 ▸). A pulsed neodymium-doped yttrium lithium fluoride (Nd:YLF) laser (527 nm, ∼3 mJ per ∼150 ns pulse, 120 Hz) was focused to a ∼0.8 mm spot within the aerosol stream, and scattered light was observed through the in-line microscope available at CXI [Questar long distance microscope, model QM-1 MK III, numerical aperture (NA) = 0.05 at 750 mm objective distance]. Images were recorded using an OPAL-4000 CCD camera and stored at 30 Hz. Fig. 1 ▸(*c*) shows a 3.7 min time-averaged optical image of particles exiting the injector. We determined that particles moved at speeds of approximately 300 m s^−1^ when they exited the injector, to arrive at the X-ray interaction point within a flight time of less than 1 µs. This particle speed was evaluated from the streak length of recorded particle images produced by laser illumination with a known pulse duration, conducted during laboratory characterization of the CNAI (see Fig. 2*a* and §3). The CNAI tip is seen to the left of Fig. 1 ▸(*c*), and the approximate X-ray focal point is indicated by the star. The particle stream could not be observed at points close to the CNAI tip because direct scattering from the tip saturated the imaging CCD.

## 3. Injector characterization and hit-fraction estimates   

In order to develop and characterize the operation of the CNAIs we conducted tests of both 15 and 30° CNAIs in our laboratory. The setup differed from our previous work (Kirian, Awel *et al.*, 2015 ▸) by the inclusion of a narrow particle transport tube intended to replicate the delivery system used at CXI. Aerosolized GV particles were transported from the nebulization chamber to the CNAI tip using stainless steel tubing of 4 mm inner diameter and 700 mm length. The GDVN was operated at flow rates of 2.7 µl min^−1^ and 28 mg min^−1^ for liquid sample and helium, respectively. A GV concentration of approximately 1.6 × 10^9^ particles ml^−1^ was used (this was diluted by a factor of 200 from the solution that was used in the CXI experiment). This flow rate and sample concentration correspond to the generation of drops at a rate of approximately 1.1 × 10^7^ s^−1^ and an entrance rate of aerosolized particles of 72 × 10^4^ s^−1^.

The imaging setup used for visualizing particles was described in detail previously (Awel *et al.*, 2016 ▸). Briefly, it was composed of an Nd:YLF laser (Spectra Physics Empower ICSHG-30, 527 nm, approximate pulse duration 100 ns, repetition rate 1 kHz, pulse energy 20 mJ) to illuminate particles, a high-frame-rate CMOS camera (Photron SA4) and a 5× magnification, 0.14 NA microscope objective to record images. The laser beam was collimated to a 2 mm spot, such that it illuminated particles across the entire field of view of the camera. The camera exposure time was set to 20 ms, such that each frame contained 20 pulses of the 1 kHz Nd:YLF laser illumination. A single image of particles emerging from the CNAI is shown in Fig. 2 ▸(*a*). The images are streaked owing to the high speed of the particles, and the observed intensity profile of these streaks reflects the relatively fast rise and slow decay of the Nd:YLF laser pulse. Centroid positions of individual particle streaks contained in 23 500 frames were used to produce the rate-corrected two-dimensional particle density map shown in Fig. 2 ▸(*b*). This rate-corrected density has units of particles per area per particle generation rate and is defined as 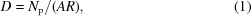
where 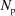 is the average number of particles that fall within a spatial bin of area *A*, and *R* is the rate at which particles entered the injector. Note that 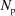 represents the average particle counts at an instant in time and not a time integration over many exposures, which is appropriate because we intend to use the particle injector with femtosecond pulses. In our case, 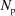 was computed by summing the number of particles that fell within each spatial bin, and then dividing by the number of recorded images and the number of laser illumination pulses per image.

The measured rate-corrected density *D* may be used to estimate the optimal hit fraction that could be achieved under idealized conditions in our X-ray measurements. If an entrance rate of 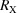 is used in the X-ray measurements, the two-dimensional particle number density is 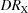. We define the effective cross-sectional area σ such that the average number of particles intercepted by an X-ray pulse is 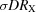. Assuming Poisson statistics, the probability of intercepting just one particle in an X-ray pulse is 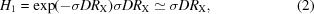
where the approximation holds to within ∼10% error as long as 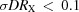. We define the X-ray beam diameter as 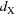 and the particle beam diameter as 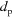 and estimate two limiting cases for the effective cross-sectional area. The first case, 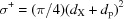, describes the optimistic limit in which a particle at the periphery of the X-ray beam produces acceptable diffraction. The second case, 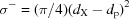, corresponds to the stronger assertion that an acceptable diffraction pattern requires that the entire X-ray beam width falls within the particle (if 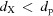) or that the entire particle falls within the X-ray beam width (if 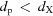). Finally, we arrive at two limiting hit-fraction estimates: 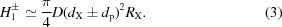



The maximum rate-corrected particle density recorded in the laboratory, *i.e.* at the focus of the particle beam shown in Fig. 2 ▸(*b*), was 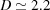 × 10^−9^ µm^−2^ s. Assuming the approximate values 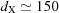
 nm, 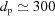
 nm and 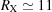
 × 10^6^ s^−1^ suggests that the maximum hit fraction to be expected in our XFEL diffraction measurements is in the range 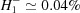 to 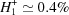. This predicted hit fraction is much higher than the hit fraction we achieved during the CXI experiment, as discussed in the next section.

## 4. X-ray diffraction analysis and discussion   

Diffraction measurements were conducted at a photon energy of 8 keV and an estimated average pulse energy of 4.2 mJ prior to the ∼30–50% beamline transmission losses (Boutet, 2016 ▸). The CSPAD detector was located 127.9 mm downstream from the X-ray focus. We recorded detector data frames for every X-ray pulse, at a rate of 120 Hz, for a cumulative total of 1.3 h, which resulted in approximately 560 000 data frames.

In all of our diffraction analysis we excluded all pixels from each detector frame that had abnormally high or low variances or mean values in ‘dark’ measurements made without X-rays, as well as a few patches of pixels for which there was obvious stray-light background. For every frame, the dark measurement was subtracted, and then a uniform common-mode electronic noise constant was subtracted from each detector panel. The common-mode offset was determined from unbonded detector pixels that are not sensitive to X-rays. The detector gain relating detector digital units to photon counts per pixel was obtained from a histogram of the pixel values, which yielded clear peaks corresponding to counts of zero, one and two photons. Most of this analysis was performed using the Python *psana* package provided by LCLS (Damiani *et al.*, 2016 ▸).

**Figure 3:**
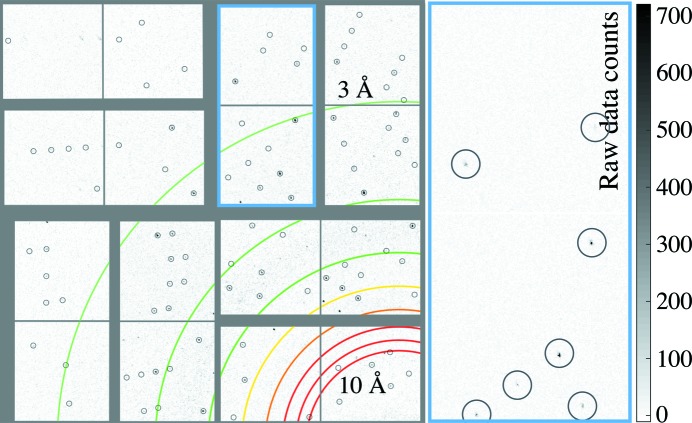
One detector quadrant of an indexed diffraction pattern obtained from aerosolized GV crystals. The colored rings indicate the resolution from 10 to 3 Å, in steps of 1 Å. The gray circles in the left-hand panel indicate the expected locations of Bragg peaks as determined by auto-indexing in the *CrystFEL* software suite (White *et al.*, 2012 ▸, 2016 ▸). The right-hand panel shows an expanded view of an individual detector tile, marked by the blue rectangle on the left. Circles in this expanded-view panel indicate peaks that are easily recognizable by eye. Notably, the predicted peak locations indicated by *CrystFEL* do not perfectly agree with those that the human eye notices, but this is typical of first indexing results and could be improved through the *CrystFEL* post-processing routines.

Fig. 3 ▸ shows one quadrant of a recorded diffraction pattern from an aerosolized GV crystal, where the average detector dark frame and common-mode offsets have been subtracted. A total of 33 hits from GV were recorded, corresponding to a hit fraction of ∼0.006%. Twenty-four patterns (73% of hits) were indexed using the *CrystFEL* software suite (White *et al.*, 2012 ▸, 2016 ▸). Autoindexing failed on patterns that appeared to consist of multiple crystals clumped together. We expect the hit fraction for our aerosol injector to be significantly lower than a typical liquid jet (about 1–10%) because of the ∼25-fold higher particle speed of the aerosol beam and the approximately fourfold reduction in liquid flow rate. However, our recorded hit fraction was still lower than the range 0.04–0.4% that we estimated from our laboratory measurements.

For comparing the background obtained using the CNAI with that typically observed in liquid-jet experiments we examined data from a previous SFX experiment (Oberthuer *et al.*, 2017 ▸) in which the exact same GV sample was injected into the X-ray beam as a liquid suspension with a GDVN. All experimental parameters were identical in both the CNAI and GDVN measurements except for the pulse energy, which was 4.6 mJ on average for the GDVN measurements.

**Figure 4:**
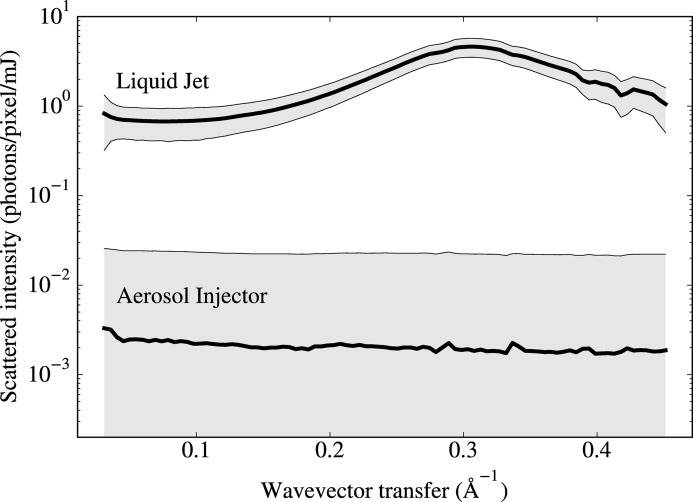
Average radial intensity profiles, on a logarithmic scale, for data measured using the GDVN (labeled ‘Liquid Jet’) and the CNAI (labeled ‘Aerosol Injector’) injectors. The average per-pixel standard deviations determined from more than 10 000 frames are indicated by the vertical width of the gray regions. After averaging, the profiles and standard deviations were normalized by dividing by the average pulse energy, and then divided by the digital-to-photon conversion factor of 18.3. The horizontal axis corresponds to the wavevector transfer 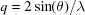, where θ is the Bragg angle and λ is the wavelength.

The comparison of background scattering for the CNAI and GDVN approaches is presented in Fig. 4 ▸, which shows a plot of the normalized azimuthally averaged profiles of scattered-photon counts (per pixel and per mJ of pulse energy), as a function of photon-wavevector transfer. The per-pixel standard deviations in the measurements are indicated by the gray regions in Fig. 4 ▸. The average profiles were divided by the average pulse energy to account for the slightly higher pulse energy in the case of the GDVN. The frames used in Fig. 4 ▸ were sampled uniformly from the final ∼5 min of data collection, when the conditions were closest to optimal, although little difference was noticed in other measurement segments. We excluded frames that fell below 1 mJ pulse energy. We additionally excluded frames that were visually corrupt as well as those for which the X-rays obviously missed the liquid jet, which corresponded to less than 10% of the frames. After removing these outliers, we confirmed that more than 10 000 frames contributed to each of the two profiles.

As can be seen from the plots in Fig. 4 ▸, the liquid jet produces a background that is over 1000 times higher at a wavevector transfer of 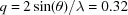
 Å^−1^, corresponding to a resolution of 3.1 Å, where θ is the Bragg angle and λ the wavelength. This coincides with the mean distance between oxygen atoms in water where diffuse scattering from water has its maximum. At low scattering angles the background from the liquid jet was about 200 times higher than for aerosol injection. The liquid jet for these measurements was operating at a flow rate of 20 µl min^−1^. Typical liquid flow rates needed to produce a stable jet range of 5–30 µl min^−1^, depending on the viscosity and surface tension of the liquid and the nozzle geometry. The volume of liquid that interacts with the X-ray beam scales roughly as the square root of the volumetric flow rate (Beyerlein *et al.*, 2015 ▸), and thus the liquid-jet background is rather typical.

Because we use a convergent micro-focused particle beam, the hit fractions are highly sensitive to the relative positioning of the CNAI with respect to the X-ray beam. Our initial diagnostic for particle beam positioning was direct imaging of scattered light, which allowed for the rough positioning of the CNAI. From this initial position, it was necessary to perform a subsequent two-dimensional scan of the injector position in an effort to optimize the spatial overlap between particle beam focus and X-rays. Owing to the limitations of our 6 h measurement shift, we only performed one 200 µm scan in the direction transverse to the particle beam and one 400 µm scan along the particle beam direction. It is therefore highly unlikely that we located the ideal position that maximizes the hit fraction. However, we expect that the background scatter we observed is representative of the gas and water vapor exiting the injector because the gas expansion into vacuum is highly divergent. Direct imaging of the gas density leaving the CNAI (Horke *et al.*, 2017 ▸) shows that the gas plume spans a volume hundreds of micrometres wide around the XFEL beam position.

Another possible culprit for our sub-optimal hit fraction is a sub-optimal aerosol transmission efficiency, which might be remedied by reducing the overall transportation tube length, by increasing the particle generation rate, by decreasing the particle speed, by increasing the volumetric flow rate of carrier gas or by the addition of aerodynamic lenses within the transport tube, which would maintain particles near the center of the transport tube. Although aerosol injection hit fractions tend to be relatively low in comparison to liquid jets, recent work at the CXI instrument reported hit fractions of 0.83% for aerosolized 40 nm viruses delivered with an aerodynamic lens stack aerosol injector (Daurer *et al.*, 2017 ▸).

Although it is convenient that our miniaturized CNAI is compatible with standard GDVN mounting hardware, the downside is that the small exit aperture, 100 µm diameter in our case, is prone to clogging. We have successfully operated our CNAIs in the laboratory for many hours without interruption, but clogging typically occurs whenever the aerosolization liquid jet misbehaves and produces large droplets for a period of a few minutes. It is, therefore, essential to ensure the formation of small droplets and continuous flow of carrier gas. In the XFEL experiment reported here, there were a total of three clogged aerosol nozzles, each of which required ∼20–30 min to replace. The severity of this issue could be greatly reduced by filtering out large droplets with, for example, an in-line impactor (Maenhaut *et al.*, 1996 ▸), and by using electrospray ionization to produce smaller initial droplet diameters (Yamashita & Fenn, 1984 ▸; Chen *et al.*, 1995 ▸; Bogan *et al.*, 2008 ▸).

It must finally be noted that the GV crystals utilized here are notoriously robust and survive in nearly pure water. For crystals that dissolve, for instance, upon varying pH, it may be feasible to avoid droplet evaporation by using a humidified carrier gas, by using electrospray nebulization or by simply placing the nebulization source close to the entrance of the aerosol nozzle to reduce the time of transport.

## 5. Conclusions   

We have demonstrated X-ray diffraction from aerosolized sub-micrometre protein crystals with background levels drastically lower than in typical SFX experiments utilizing liquid jets. This may be important for coherent-diffractive-imaging experiments on weakly scattering targets such as isolated proteins, viruses or cells, as well as for the measurement of diffuse scattering or lattice-transform signals between crystalline Bragg reflections (Ayyer *et al.*, 2016 ▸; Kirian, Bean *et al.*, 2015 ▸). We showed that our injector is compatible with the existing hardware at LCLS, allowing quick changes from a liquid jet to an aerosol injection system in a single experiment. The relatively high (∼300 m s^−1^) particle speeds may be useful for avoiding damage due to X-ray-induced explosions when using new XFEL sources with pulse repetition rates up to 4.5 MHz.

While the obtained 0.006% hit fraction at LCLS was much lower than in typical liquid-jet X-ray diffraction experiments, laboratory measurements suggest that this can be improved by orders of magnitude. On the basis of these laboratory measurements, we suspect that the low hit fractions observed in this study are a result of aerosol transport losses, clustering of particles, clogging of the aerosol nozzle due to an under-performing GDVN nebulizer, or misalignment between the X-ray focus and particle beam focus. As we have noted, there are several possible routes to improve upon the injection strategy described here, as shown by other aerosol injection work performed at the same CXI instrument (Daurer *et al.*, 2017 ▸).

Above all, the lower background achieved with the aerosol nozzle somewhat offsets the lower hit fraction, since the number of required measurements depends inversely on the square of the signal-to-noise ratio of intensities, or is directly proportional to the background counts.

This proof-of-principle experiment was performed on granulovirus occlusion bodies suspended in water. These protein crystals have naturally evolved to be robust against the change in the buffer conditions and dehydration caused by evaporation of the liquid layer on the crystals’ surface. However, most protein crystals are not stable in pure water. When working with other types of crystals, the liquid buffer evaporation rate on the surface of the crystals must be controlled, for example by controlling the relative humidity at the crystals (Sanchez-Weatherby *et al.*, 2009 ▸; Roedig *et al.*, 2015 ▸).
